# Comprehensive diagnostic and therapeutic approach to male factor infertility aimed at natural fertility: A multicentric retrospective cohort study

**DOI:** 10.1111/andr.70006

**Published:** 2025-02-10

**Authors:** Giuseppe Grande, Andrea Garolla, Andrea Graziani, Anna Laura Astorri, Maria Vittoria Cammarota, Annamaria Merola, Maria Pia Polidori, Emanuela Lulli, Enrico Busato, Francesco Pesce, Giuseppina Pompa, Alfredo Pontecorvi, Domenico Milardi, Alberto Ferlin

**Affiliations:** ^1^ Unit of Andrology and Reproductive Medicine, Department of Medicine University of Padova Padova Italy; ^2^ Division of Endocrinology Centre for Natural Reproductive Medicine “ISI ‐ Paul VI”, Fondazione Policlinico Universitario “Agostino Gemelli” Scientific Hospitalization and Treatment Institute (IRCCS) Rome Italy; ^3^ Foundation “Don Luigi Saccone Pozzuoli (NA) Italy; ^4^ Department of Women's, Children's and Public Health Sciences Fondazione Policlinico Universitario “Agostino Gemelli” Scientific Hospitalization and Treatment Institute (IRCCS) Rome Italy; ^5^ Clinical Centre “St. Joseph” Lucrezia di Cartoceto (PU) Italy; ^6^ Obstetrics and Gynaecology Department Ca' Foncello Hospital Treviso Italy; ^7^ Fondazione Centro della Famiglia, Counseling Center Treviso Italy

**Keywords:** couple infertility, idiopathic infertility, male factor infertility, natural fertility, semen analysis

## Abstract

**Background:**

In infertile couples whose male partner has alterations in semen parameters frequently, a comprehensive andrological approach is lacking and approximately 30–50% are classified as idiopathic infertility. These couples are often directly addressed to assisted reproduction techniques (ARTs). However, several clinical conditions may benefit from medical treatment. By acting on etiology and/or risk factors, this aims at improving seminal parameters and restoring natural fertility.

**Objectives:**

To verify the impact of a comprehensive andrological assessment on the management of infertility (in particular, in couples with isolated male factor infertility) using as the primary outcome the natural pregnancy rate.

**Materials and methods:**

A multicenter retrospective study was conducted between 2015 and 2022 in 1014 couples with primary infertility seeking natural conception (including 266 couples with previous ART failure). Each couple underwent a multidisciplinary evaluation. This involved: a gynecologist and an andrologist both with expertise in infertility, a psychologist when requested, and a fertility awareness practitioner according to a unique diagnostic and therapeutic multidisciplinary protocol.

**Results:**

An isolated male factor was found in 23% of couples. In 45%, it was associated with female factors also. The comprehensive diagnostic approach reduced the proportion of idiopathic infertility to 8% of the couples. Targeted treatment, based on diagnostic categories, was associated with spontaneous pregnancy in 40.9% of the couples. In the 233 cases without female factors, normal semen parameters were observed only in 13% of patients. Male genital tract inflammation was observed in 48.8% of the patients, genital tract infection in 43.1%, and hypospermatogenesis in 16.7%. Patients with infections were treated with antibiotics and probiotics. If further inflammation was documented, this was followed by low‐dose corticosteroids and antioxidants. Follicle stimulating hormone (FSH) treatment was used in patients with hypospermatogenesis, and varicocele repair surgery was performed in four patients.

**Discussion and conclusions:**

Our data underline the efficacy of a comprehensive approach to the diagnostic process of male factor infertility, both in reducing the percentage of idiopathic infertility and in restoring natural fertility based on a targeted treatment.

## 1 INTRODUCTION

Infertility is a common clinical condition. It is defined by the lack of conception after at least 12 months of regular unprotected sexual intercourse.^1^ About 10–15% of couples worldwide are infertile, with 56% of them seeking medical care.^2^ Male factor infertility contributes to 30–50% of infertility cases, representing approximately one in 20 men in Western society.^2^, ^3^, ^4^ While poorly quantified, at a social and financial level, male factor infertility is a highly significant medical and social problem. It contributes to the growing use of assisted reproduction technology (ART) and substantial costs to individuals and the healthcare system.^5^

Despite this, 30–50% of the male partners of infertile couples who have alterations in semen parameters are classified as idiopathic (or unexplained) infertility^6^, ^7^—often directly addressed with ART.

Moreover, it has been demonstrated that approximately 15% of couples undergo ART without any prior andrological evaluation. This trend has been confirmed over most recent years.^8^

Over the past two decades, there have been several concerns about the overuse of ART^9^ because of the lack of evidence regarding effectiveness in certain populations (e.g., unexplained infertility),^10^ potential short‐ and long‐term safety,^11^, ^12^ and economic considerations.^13^ Furthermore, for some patients, ART might raise ethical problems. This should be considered to safeguard the patient−doctor relationship and to respect the patient's autonomy to make mature and responsible choices.^14^

In clinical practice, it is common to meet infertile couples who request medical evaluation and treatment aimed at the restoration of natural fertility, while avoiding the use of ART.^15^ Some protocols have been actioned by shifting to a modern multidisciplinary care pathway, including medical evaluation (andrological and gynecological), fertility awareness, and psychological support.^16^

In the area of male factor infertility, the Italian Society of Andrology and Sexual Medicine (endorsed by the Italian Society of Embryology, Reproduction, and Research) has recently proposed some evidence‐based recommendations for the diagnosis, treatment, and management of male factor infertility to improve patient and couple care.^17^ These guidelines are based on two principal aspects: they are couple‐oriented, and they place high value on assessing, preventing, and treating the risk factors for infertility.

Starting from these premises, we performed a retrospective multicenter study on couples seeking natural fertility. We aimed to analyze the impact of a comprehensive diagnosis and targeted medical treatment of infertility using as an outcome the natural pregnancy rate, in particular in couples with isolated male factor infertility.

## 2 MATERIALS AND METHODS

A multicenter, retrospective study was conducted between 2015 and 2022 in 1014 couples with primary infertility seeking natural conception. Patients were evaluated at the infertility clinics at the Centre for Natural Reproductive Medicine “ISI Paul VI” at the Fondazione Policlinico Universitario A. Gemelli IRCCS, Rome, at the Counseling Centre of “Centro della Famiglia” Foundation in Treviso, at the “Centro Medico S. Giuseppe” in Lucrezia di Cartoceto (PU), and at the “D. Luigi Saccone” Foundation in Pozzuoli (NA), Italy.

The study protocol follows the standard clinical approach and the principles outlined in the Declaration of Helsinki. The study has been performed following the Italian ethical rules as considers retrospective studies of patient data.

### 2.1 Patients

All couples were unable to conceive for at least 12 months. Each couple underwent a multidisciplinary evaluation with: a gynecologist and an andrologist with expertise in infertility, a psychologist (when requested), and a fertility awareness practitioner. Patients were assessed according to a unique standardized diagnostic and therapeutic multidisciplinary protocol. Informed consent for this protocol was expressed, followed by signing to refuse to undergo ART. The informed consent has been approved by the Institutional Review Board of the International Scientific Institute “Paul VI” at the Catholic University in Rome.

For this study, we evaluated only couples with a minimum follow‐up period of 12 months.

### 2.2 Diagnostic and therapeutic protocols

The female partners underwent history collection, physical examination, hormonal assessment of ovulatory function, screening for cervical and vaginal infections, and transvaginal sonogram according to current guidelines.^3^ Evaluation of tubal patency was performed by sonohysterography or by hysterosalpingogram in women with normal ovarian reserve and without endocrine diseases or in women with polycystic ovary syndrome (PCOS) before ovulation induction. Antibiotic treatment was given to patients with documented infections on cervical and vaginal swabs. Patients with chronic anovulation due to PCOS were treated according to current guidelines^18^ using Letrozole. Patients with septate and arcuate uterus were treated by means of operative hysteroscopy, performed with cold scissors or electrosurgery. Tubal reconstructive surgery was performed, in case of tubal disease, when possible, as previously reported.^19^ Proximal tubal occlusion was treated using a microsurgical approach by laparotomy as previously reported.^20^ Operative laparoscopy has been performed for salpingo‐ovariolysis in patients with periadnexal adhesions and a normal tubal mucosa.^21^ Laparotomic salpingoneostomy was performed using microsurgical techniques in women with distal tubal occlusion.^22^ Surgical excision of moderate–severe endometriosis has been proposed in women with infertility and endometriotic ovarian cysts (stage III/IV), after informing the patients that the effect of endometrioma excision on reproductive prognosis is poorly defined and that the chances of conception without surgery are currently unclear, as reported in current guidelines.^23^

Finally, all women were instructed on natural family planning methods to identify the fertile window.

The male partners underwent history collection, physical examination, standard semen analysis according to WHO guidelines,^24^ and scrotal ultrasound. Endocrine factors, non‐endocrine testicular dysfunction, and genital tract infections and inflammations were also evaluated, according to guidelines.^3^, ^17^

Patients underwent two semen analyses (within 3 months of each other), endocrine assessment of testicular function, and other hormonal diseases impacting on fertility included Follicle stimulating hormone (FSH), Luteinizing hormone (LH), total testosterone, estradiol, Sex hormone binding globulin (SHBG), albumin, vitamin D, Prolactin (PRL), Thyroid stimulating hormone (TSH), and scrotal ultrasound. Semen analysis followed the indication included in the checklist that has been proposed for studies that include basic semen analysis examinations.^25^

Male tract infection was suspected in the presence of symptoms and/or seminal alterations such as leukocytospermia and/or alterations in semen pH and/or viscosity, or positivity of anti‐sperm antibodies. In these cases, semen culture and nucleic acid amplification tests via urethral swabs (for Chlamydia trachomatis and Mycoplasma) were sent off. Transrectal ultrasound prostate‐vesicular scan was performed in patients with suspected genital tract infection and inflammation.

Diagnosis of male genital tract inflammation was done in the presence of both positive seminal findings (leukocytospermia, alterations in semen pH and/or viscosity, positivity of anti‐sperm antibodies) and ultrasonographic signs of inflammation, as previously reported.^26^, ^27^ We defined signs of inflammation on ultrasound as follows:

‐ For the epididymis, >2 of the following ultrasonographic signs: a. increase in size of the head and/or of the tail; b. presence of multiple microcystis in the head and/or tail; c. low or high echogenicity; d. large hydrocele mono or bilateral.
‐ For the prostate, >2 of the following ultrasonographic signs: a. asymmetry of the gland volume; b. areas of low echogenicity; c. areas of high echogenicity; d. dilatation of peri‐prostatic venous plexus; e. single or multiple areas of acinar ectasia; f. area/s of moderate increased of vascularity (focal or multiple).
‐ For the seminal vesicles, >2 of the following ultrasonographic signs: a. increase of anteroposterior diameter; b. asymmetry >2.5 mm compared to the controlateral vesicle; c. glandular epithelium thickened and/or calcified; d. polycyclic areas separated by hyperechoic septa in one or both vesicles; e. fundus/body ratio >2.5 or fundus/body ratio <1.

Testicular fine needle aspiration cytology was performed in cases of azoospermia and severe oligozoospermia (<5 mill. sperm/mL) and candidates to FSH treatment.^28^

Genetic assessment was performed according to guidelines,^29^ which included: a karyotype if sperm concentration was <10 mill./mL, testing for Yq microdeletions when sperm concentration was <5 mill./mL, and testing for mutations of the CFTR gene in patients with congenital uni‐ or bilateral absence of vas deferens.

Couples were considered to have idiopathic infertility in cases of apparent normal female factors (ovarian function, fallopian tubes, uterus, cervix, and pelvis, age <40 years), normal male factors (testicular function, genito‐urinary anatomy, and a normal ejaculate), and adequate coital frequency.^30^ As a consequence, we defined a couple as affected by idiopathic infertility when no significant alterations have been observed both in the woman (normal hormonal assessment of ovulatory function of woman, negative screening for cervical and vaginal infections, normal transvaginal sonogram, documented tubal patency) and in men (normal semen analysis, normal hormone assays, negative screening for male tract infections, normal scrotal and transrectal ultrasound).^7^

Etiological treatments were performed when a causal factor was identified. Antibiotic treatment was given to patients with male genital tract infections documented following sperm culture or urethral swab^31^; FSH treatment (150 IU three times per week for 3–4 months) was used in oligozoospermic patients with normal FSH plasma levels (<8 IU/L) and hypospermatogenesis at testicular cytologic analysis.^32^ Prednisone was administered at a dose of 25 mg/day for 30 days in patients with documented genital tract inflammation, as previously reported.^33^ Antioxidants (L‐carnitine 1000 mg daily, N‐acetyl cysteine 600 mg daily, Coenzyme Q10 200 mg daily, Zinc 15 mg daily, Selenium 100 µg daily, Folic acid 400 µg daily for 3 months) were used alone in idiopathic infertility or associated with prednisone in patients with abacterial or post‐infective inflammation.^34^ Varicocele repair surgery was performed in patients with severe varicocele (grade III‐V Sarteschi classification) and in the absence of any other causes of testicular impairment or genital tract dysfunction.^35^

### 2.3 Data analysis

The prevalence of male and female factors, prevalence of idiopathic infertility, and pregnancy rate in the studied population were analyzed.

Furthermore, we analyzed the sub‐group of couples isolated male factor infertility and calculated the prevalence of seminal alterations, the relative prevalence of the different treatable clinical conditions (male genital tract infection, male genital tract inflammation, hypospermatogenesis, varicocele, idiopathic infertility), and the relative pregnancy rates.

We finally divided our population into two study groups: previous history of ART failure or no previous history of ART failure. Clinical, seminal, and hormonal characteristics were compared in the two groups. Results have been reported as: average ± standard deviation. Statistical analysis has been carried out with SPSS v18.0 (IBM Corp.). All data have been first analyzed for normality of distribution using the Kolmogorov–Smirnov test of Normality. The appropriate parametric test (*t*‐test) was used to assess the significance of the differences between groups. A *p*‐value < 0.05 was considered significant. Spontaneous pregnancy rate was evaluated in the two studied groups.

## 3 RESULTS

### 3.1 Full population of infertile couples and general pregnancy rate

The average age of the male population was (mean ± SD) 37.7±5.8 years, and of the female population was 33.4±5.5 years. The mean duration of infertility was 3.1±3.3 years.

Isolated female factor infertility has been documented in 243 couples (24%), while isolated male factor infertility in 233 couples (23%). Combined male and female factors have been identified in 456 couples (45%). Therefore, idiopathic couple infertility was diagnosed only in 82 couples (8%).

The prevalence of the different etiologies in couples with an isolated female factor is shown in Figure S1.

In 95 couples (9.4%), the identified cause of infertility was due to secretive azoospermia, premature ovarian failure, or genetic abnormalities. It was, therefore, not possible to offer these couples any therapeutic option aimed at natural conception.

**FIGURE 1:**
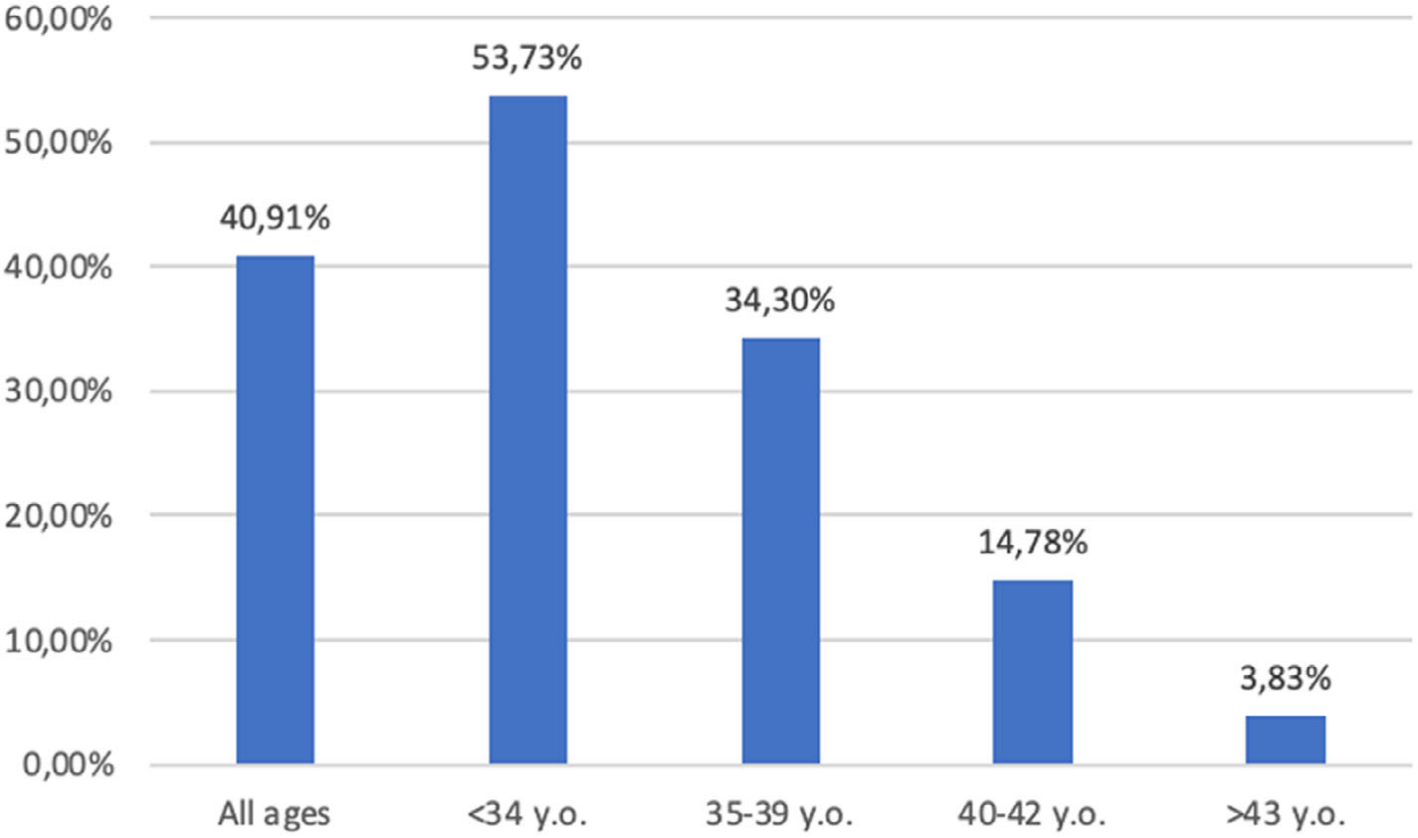
.

We have, therefore, focused our attention on the remaining 919 couples. A spontaneous pregnancy was achieved in 376/919 treated couples (40.9%). Figure 1 reports the pregnancy rate stratified for the age of the woman.

### 3.2 Couples with isolated male factor infertility

**FIGURE 2:**
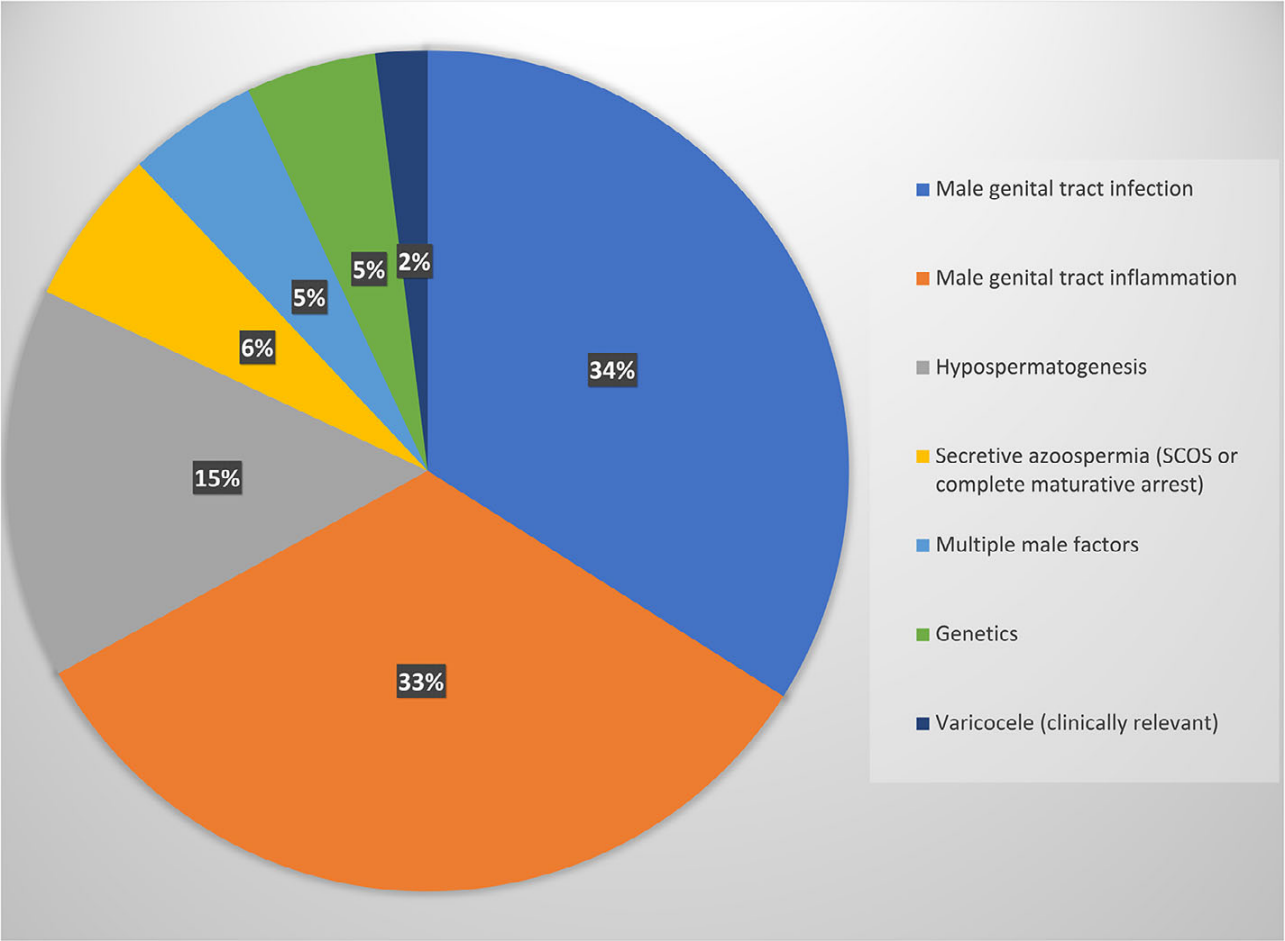
.

To analyze in detail the impact of a comprehensive diagnostic and therapeutic protocol for male factor infertility, we studied the 233 couples in which a female factor has been excluded. The different etiological causes of male factor infertility are reported in Figure 2. Importantly, untreatable causes of male factor infertility (such as secretive azoospermia due to Sertoli cell only syndrome, or complete germinal maturative arrest, and/or genetic diseases such as karyotype alterations, Y‐chromosome microdeletions, and CFTR mutations causing secretive azoospermia) were found in only 24 patients.

After a detailed diagnostic approach, 209 patients were treated according to current clinical guidelines and position statements of scientific societies, based on the specifically identified etiology of their infertility.^3^, ^17^

Sperm parameters (sperm count, motility, morphology) >5th percentile values were observed only in 13% of patients, while different alterations in sperm parameters were found in the remaining 87%. Patients with sperm parameters >5th percentile, included in this group, had, however, symptoms and/or seminal alterations (leukocytospermia, alterations in semen pH and/or viscosity, or positivity of anti‐sperm antibodies) suggestive of male tract infection and/or ultrasound signs of inflammation at epididymis, so that they performed the complete diagnostic workflow for male tract infections/inflammation.

We used a novel classification for male infertility factors using these etiological classes: male genital tract infection; male genital tract inflammation; hypospermatogenesis; genetic alterations; varicocele; and secretive azoospermia.

Spontaneous pregnancy has been achieved in the group of patients with isolated male factor infertility in 68/209 patients (32.5%). Table 1 reports each treated clinical condition and the relative pregnancy rate after treatment.

**TABLE 1:** Pregnancy rate for each group of male patients, according to the diagnosis.

Clinical condition	Spontaneous pregnancy (*n*; %)
Abacterial male genital tract inflammation	17.6%
Male genital tract infection	47.8%
Hypospermatogenesis	17.1%
Varicocele	25.0%

### 3.3 ART failure vs. “naïve” couples

Among the 919 treated couples, 28.9% (*n* = 266) had previous ART failure (with a mean of 2.3 cycles per couple). Clinical, seminal, and hormonal data of the two sub‐groups (ART‐failure and non‐ART‐failure) are reported in Table 2. Patients in the ART failure group had a significantly higher female age, a higher time of infertility, and a significantly reduced testicular volume.

**TABLE 2:** Comparison between clinical, seminal, and hormonal data in treated couples with and without previous ART failure.

	Non‐ART‐failure group (*N* = 653)	ART‐failure group (*N* = 266)
Age (male) (year)	37.14±5.72	39.3±5.9
Age (female) (year)	33.57±6.07	35.4±4.7^*^
Time of infertility (year)	2.42±2.81	4.9±3.9^*^
Bitesticular volume	26.3±8.23	22.06±11.27^*^
Semen volume (mL)	4.1±3.20	3.56±1.53
Semen pH	7.92±0.67	7.9±0.34
Sperm concentration (x106/mL)	24.28±34.17	17.34±18.54
Total sperm count (x10^6^/ejaculate)	34.21±28.04	38.95±15.22
Total sperm motility (%)	12.48±16.95	15.5±13.05
Normal sperm morphology (%)	8.13±17.95	4.25±3.86
FSH (IU/L)	6.9±5.8	6.07±4.29
LH (IU/L)	4.9±2.4	3.7±1.52
Testosterone (ng/mL)	4.9±1.9	5.4±2.2
Pregnancy rate (%)	40.7%	36%

Abbreviations: FSH, Follicle stimulating hormone; LH, Luteinizing hormone.

* *p*<0.05.

In couples without a history of ART failure, a spontaneous pregnancy was observed in 280 couples (42.9%). In the population with previous ART failure, the spontaneous pregnancy rate was 36.1% after infertility treatment.

## 4 DISCUSSION

Despite decades of research, the etiology of infertility remains often poorly understood, and previous data reported that no cause is identified in almost 30% of cases.^36^ A single‐center prospective study was previously conducted aimed at understanding the most frequent causes of male factor infertility. The authors reported that, among 8518 patients, an etiological cause of infertility was identified in only 40% of them. In the same population, 75% of oligozoospermic patients were classified under idiopathic infertility.^37^ Similarly, another study showed that among 26,091 male patients attending an infertility center, the etiology of infertility remained unexplained in up to 72% of cases.^38^ Importantly, too often the diagnosis of idiopathic infertility is defined without a thorough diagnostic procedure.^30^ This diagnosis should instead be made only after all known causes of male factor infertility have been properly evaluated and excluded, using a detailed and meticulous diagnostic process.^7^

Here, we reported the results of an integrated multidisciplinary approach to the couple with infertility. The application of a rigorous diagnostic and therapeutic protocol demonstrated that the diagnosis of idiopathic infertility may be reduced to 8%. Although several studies report the relative prevalence of different etiologies of female factor infertility (as also reported in this study), male factor infertility is often neglected and generally ART reports refer to “male factor infertility” without stratifying the different etiologies, but referring only to seminal parameters. We demonstrated the importance of investigating the male factor in detail, since according to our data, a clinical condition associated with male factor infertility has been detected in 68% of the infertile couples, alone (23%) and associated (45%) with a female factor.

Furthermore, 13% of patients presented with “normal” semen analysis, just considering sperm parameters >5th percentile. These couples would be categorized as affected by “idiopathic infertility” if the diagnostic protocol would be stopped after semen analysis. However, semen analysis is not always and not strictly predictive of fertility.^14^ The last edition of the WHO manual for semen analysis underlines the continuum between fertile and infertile patients, confirming that no threshold in semen parameters may be given to detect and predict male fertility potential or infertility.^24^ Consequently, semen analysis (despite representing a central and pivotal examination in the evaluation of the infertile male) cannot be the only test performed in a clinical evaluation. Instead, it represents the starting point for further analysis based on history, clinical examination, and other diagnostic procedures.

A spontaneous pregnancy rate of 40.9% has been observed. As expected, the pregnancy rate declined as the age of the woman increased. However, it should be noted that in women <34 years old, the pregnancy rate is approximately 50%. In women <40 years old, it is approximately 30%. Previous data published by the registries about the use of ART in Italy and in Europe^39^ reported an overall cumulative pregnancy rate for In vitro fertilization (IVF)/Intracytoplasmicsperm injection (ICSI) using fresh gametes of 15.4% per cycle in Italy, and 25.5% per aspiration in Europe. In Italy, the pregnancy rate per cycle is higher (21.6%) for young women <34 years, then declines to 17.3% in women 35–39 years, to 10.1% in women 40–42 years, and finally to 4.1% in women >43 years. Pregnancy rates per aspiration in Europe are reported to be 30.8%, 25.4%, and 13.6%, respectively, for women <34 years, 35–39 years, and >40 years.

Our results, therefore, suggest that directly performing IVF/ICSI as the first‐line treatment of couple infertility does not lead to a significant advantage on pregnancy rate, at least for women <40 years. Consequently, our data underline, especially for younger couples, the importance of a comprehensive diagnosis and treatment approach, in a multidisciplinary and parallel way of both gynecological and andrological factors, before addressing a couple to ART.

In fact, we demonstrated a significant spontaneous pregnancy rate in couples with previous ART failure (36%). Previous data showed spontaneous conception in about 10–15% of the couples waiting for ART treatments.^40^, ^41^ Here, we reported for the first time, at the best of our knowledge, that more than one out of three couples with a previous ART failure may obtain a spontaneous pregnancy after etiological treatments of conditions associated with infertility. This evidence once again calls into question the direct address of infertile couples to ART. Especially in younger couples, a spontaneous conception might be achieved, therefore, reducing the invasiveness of the procedures and the costs for the community.

Finally, we analyzed the prevalence of the different clinical conditions associated with isolated male factor infertility and the impact of their treatment in terms of pregnancy rate. Previous data showed that up to 27% of male partners of couples seeking fertility care do not receive an andrological evaluation.^42^ Evaluation of the male often is neglected because women are considered the only “patients” in fertility treatment.^43^ Additionally, the public and provider perception of infertility is that it is primarily a gynecological problem, combined with the misconception that the use of ARTs can circumvent a male factor problem—thus unwittingly minimizing the importance of the male evaluation. Therefore, infertility is not a matter for the woman or the man alone, it is a matter for the couple, so the clinical approach should be personalized to consider different aspects specific to each couple.

We showed that, when a correct and extensive diagnostic and therapeutic process is applied for the diagnosis and treatment of male factor infertility, a spontaneous conception may be obtained in 32.5% of couples with isolated male factor infertility.

Furthermore, we analyzed the prevalence of the different clinical conditions associated with male factor infertility and proposed a novel ethiological classification based on the different clinical conditions associated with or responsible for male factor infertility (male genital tract infection; male genital tract inflammation; hypospermatogenesis; genetic alterations; varicocele; secretive azoospermia). This is a novel approach to consider male factor infertility, aimed to evaluate not the seminal abnormality—as often reported—but the clinical conditions responsible for or associated with the seminal alterations.

Male genital tract infection has been detected—alone or associated with other clinical conditions—in 43% of the infertile patients, in agreement with the published data. In fact, we already reported that male tract infections accounted for 37% of patients with a spontaneous pregnancy after fertility treatments.^14^ More recently, Olana et al. reported that the prevalence of positive semen culture in infertile couples was 43.5%.^44^ The treatment of male tract infections requires sequential treatment with antibiotics and probiotics. If a genital tract inflammation is documented, this can be followed by a transrectal ultrasound scan of the prostate and accessory vesicles, as well as a scrotal ultrasound to evaluate signs of epidydimal inflammation.^45^ Treatment may include low‐dose corticosteroids, and sequential adjuvant treatment with antioxidants, as previously reported.^46^ Previous studies demonstrated the persistence of altered seminal parameters and ultrasound signs of inflammation in 18% of patients treated with antibiotics for genital tract infections with bacterial eradication.^47^ We reported, for the first time, the efficacy of this sequential and global approach, resulting in a pregnancy rate of 17.8%.

Furthermore, we previously reported the efficacy of prednisone treatment for patients with abacterial or post‐infective male genital tract inflammation^33^ in improving seminal parameters (namely, sperm count and motility). Here, we demonstrated, for the first time, the efficacy of the same protocol of treatment, in terms of spontaneous pregnancy rate, since 47.8% of the treated patients obtained a spontaneous conception.

Several data demonstrated the efficacy of FSH treatment in patients with spermatogenesis impairment and normal FSH levels, both in terms of improvement in seminal parameters and of spontaneous and assisted pregnancies.^48^, ^49^ FSH treatment increases spontaneous pregnancy rate, with an overall odds ratio of about 4.5.^49^ Spontaneous pregnancy rate in FSH‐treated patients has been previously estimated at about 9%,^48^ ranging from 8.8%^50^ to 10.5%.^51^ Furthermore, previous studies suggested that testicular cytology assessment by fine needle aspiration of the testis may be important in predicting the response to FSH treatment.^52^ FSH, in fact, increases the spermatogonial population and sperm production in patients with oligozoospermia with normal FSH and a cytological picture of hypospermatogenesis, whereas the efficacy of FSH treatment is lower in patients with oligozoospermia and maturation disturbances of spermatogenesis.^32^ The results obtained in the present study on patients with hypospermatogenesis confirm previous suggestions, underlining that a high spontaneous pregnancy rate (17.1%) may be obtained if we select the patients to be treated with FSH by testicular cytology.

Finally, only 8% of infertile patients had a clinically relevant varicocele (i.e., a grade III‐V varicocele without any other identifiable cause or risk factor of alteration of semen parameters). These patients underwent varicocele repair surgery, and one spontaneous pregnancy was registered among four treated patients.

The limitations of this study are represented by the absence of a control group to compare the pregnancy rate in couples directly addressed to ART.

## 5 CONCLUSIONS

This study showed the efficacy of a complete diagnostic evaluation and etiological treatment of couple infertility. This is particularly important for younger couples. It reinforces the importance of a multidisciplinary and parallel approach of both gynecological and andrological factors, as well as the need to provide treatment options when a clinical condition is diagnosed, before addressing a couple to ART. To improve the current system, we proposed a novel classification of male factor infertility oriented to the identification of treatable conditions, showing the efficacy of an etiologic‐oriented treatment.

## AUTHOR CONTRIBUTIONS

*Conception and design*: GG, AGa, and AF. *Acquisition of data*: GG, ALA, GP, and DM. *Data analysis*: GG and AGr. *Data interpretation*: MVC, AM, MPP, EL, EB, FP, and AP. *Drafting of the article*: GG, AGr, and DM. *Critical revision for important intellectual contents*: AGa, ALA, MVC, AM, MPP, EL, EB, FP, GP, AP, and AF. All authors gave final approval of the version to be published, and agreement to be accountable for all aspects of the work in ensuring that questions related to the accuracy or integrity of any part of the work are appropriately investigated and resolved.

## CONFLICT OF INTEREST STATEMENT

The authors declare no conflict of interest.

## Supporting information



Supporting information

## Data Availability

The data that support the findings of this study are available from the corresponding author upon reasonable request.
