# Supplementary figures and images for: Comprehensive diagnostic and therapeutic approach to male factor infertility aimed at natural fertility: A multicentric retrospective cohort study
Source: Andrology. 2025 Feb 10;13(8):2122–30. doi: 10.1111/andr.70006 (PMC12569740; doi:10.1111/andr.70006)

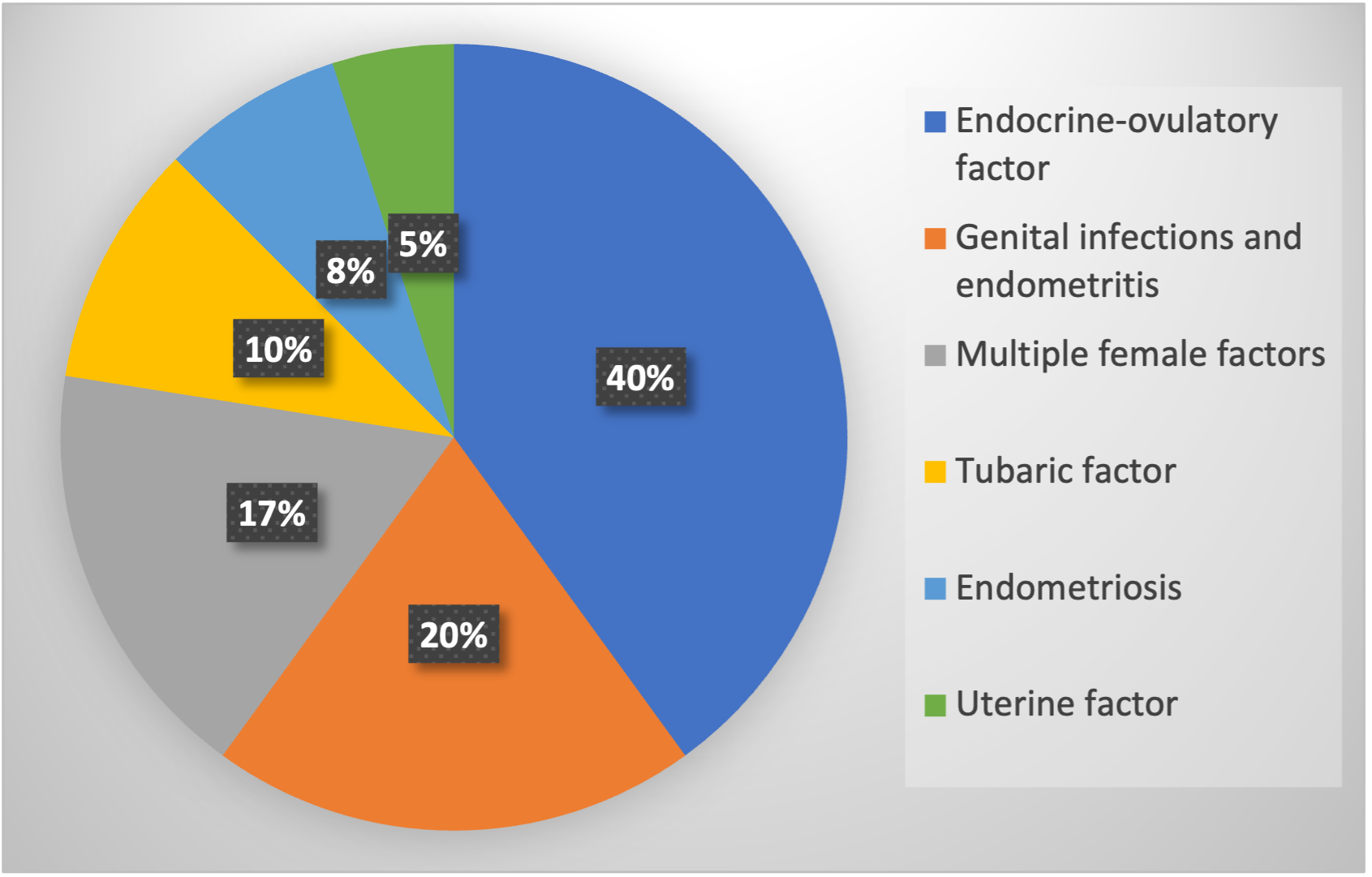

Supplement: Supplementary file 1 — Supporting information [file ANDR-13-2122-s001.png]
